# Photoreconfigurable Metasurface for Independent Full-Space Control of Terahertz Waves

**DOI:** 10.3390/s25010119

**Published:** 2024-12-27

**Authors:** Zhengxuan Jiang, Guowen Ding, Xinyao Luo, Shenyun Wang

**Affiliations:** 1 Research Center of Applied Electromagnetics, Nanjing University of Information Science and Technology, Nanjing 210044, China; 202283320060@nuist.edu.cn (Z.J.); xyluo@nuist.edu.cn (X.L.); 002429@nuist.edu.cn (S.W.); 2 School of Electronic Science and Engineering, Nanjing University, Nanjing 210093, China

**Keywords:** photoreconfigurable, full-space metasurface, terahertz, wavefront manipulation, perovskite

## Abstract

We present a novel photoreconfigurable metasurface designed for independent and efficient control of electromagnetic waves with identical incident polarization and frequency across the entire spatial domain. The proposed metasurface features a three-layer architecture: a top layer incorporating a gold circular split ring resonator (CSRR) filled with perovskite material and dual *C*-shaped perovskite resonators; a middle layer of polyimide dielectric; and a bottom layer comprising a perovskite substrate with an oppositely oriented circular split ring resonator filled with gold. By modulating the intensity of a laser beam, we achieve autonomous manipulation of incident circularly polarized terahertz waves in both transmission and reflection modes. Simulation results demonstrate that the metasurface achieves a cross-polarized transmission coefficient of 0.82 without laser illumination and a co-polarization reflection coefficient of 0.8 under laser illumination. Leveraging the geometric phase principle, adjustments to the rotational orientation of the reverse split ring and dual *C*-shaped perovskite structures enable independent control of transmission and reflection phases. Furthermore, the proposed metasurface induces a +1 order orbital angular momentum in transmission and +2 order in reflection, facilitating beam deflection through metasurface convolution principles. Imaging using metasurface digital imaging technology showcases patterns “NUIST” in reflection and “LOONG” in transmission, illustrating the metasurface design principles via the proposed metasurface. The proposed metasurface’s capability for full-space control and reconfigurability presents promising applications in advanced imaging systems, dynamic beam steering, and tunable terahertz devices, highlighting its potential for future technological advancements.

## 1. Introduction

Terahertz radiation, with frequencies ranging from 0.1 to 10 THz, lies between microwaves and far-infrared in the electromagnetic spectrum. Due to its high penetration and low energy characteristics, terahertz technology has significant applications in communication, radar, security inspection, and medical imaging. Consequently, terahertz wave manipulation technology has garnered widespread research attention globally. However, conventional materials exhibit limited electromagnetic responses in the terahertz band, restricting the development of terahertz devices and technology.

Metamaterials, constructed through micro-nano structural design, exhibit novel functionalities that transcend the limitations of traditional optical materials. Professor Pendry’s introduction of negative refraction metamaterials enables the creation of metalenses capable of surpassing the diffraction limit [1,2]. Despite their potential applications, high-performance and scalable metalenses still face significant challenges due to stringent operational conditions. In 2011, Capasso et al. introduced the concept of metasurfaces [3,4], pioneering a new direction in metamaterials research. The advent of metasurfaces has driven numerous innovative optical, terahertz and microwave applications, including ultrathin high-resolution planar lenses [5,6], three-dimensional holographic imaging technology [7,8], beam shaping techniques [9,10] (such as Bessel beams and vortex beams), and precise polarization control [11,12,13]. These advancements have unlocked unprecedented possibilities and expansive application prospects in the field of electromagnetic wave manipulation.

To broaden the application scenarios of terahertz waves, designing multifunctional metasurfaces has become imperative. Multifunctional metasurfaces can manipulate electromagnetic waves in various ways, enabling diverse functionalities within a single structure, such as focusing, steering, and splitting beams. This is often achieved through techniques such as polarization [14,15,16], frequency [17,18,19], or incidence direction multiplexing [20]. These capabilities enhance the metasurfaces’ potential applications in fields such as communication, imaging, and sensing.

Among multifunctional metasurfaces, full-space metasurfaces stand out due to their ability to independently and efficiently control both transmitted and reflected waves. Typically, many high-efficiency metasurfaces operate solely in either transmission or reflection mode, thus only manipulating one aspect of wave behavior and leaving half of the electromagnetic space underutilized. Conventional metasurfaces that manage transmitted and reflected waves often feature interdependent phase responses. Therefore, designing multifunctional full-space metasurfaces capable of independent and efficient control of both transmission and reflection modes, particularly their phase responses, has become essential. Mao et al. demonstrated a single-layer full-space metasurface design that enables independent control of reflected and transmitted circularly polarized waves through meta-structure rotation, achieving high-efficiency dual-band operation with 84.1–84.9% efficiency as a CP beam splitter at 8.3 GHz in reflection mode and 82.7% efficiency as a meta-lens at 12.8 GHz in transmission mode, providing a simplified and cost-effective approach for full-space electromagnetic wave manipulation compared to conventional multilayer designs [21]. Furthermore, Fan et al. demonstrated a bilayer geometric-phase metasurface capable of independently controlling the wavefronts of transmitted and reflected cross-polarized waves at distinct terahertz frequencies (0.6 THz and 1.67 THz), achieving high polarization conversion coefficients of 0.87 and 0.92, respectively, with full 2π phase modulation through geometric parameter optimization, enabling various functionalities including anomalous refraction/reflection, dual-band full-space cylindrical focusing, and multi-mode vortex beam generation in both transmission and reflection modes [22]. However, achieving independent control of electromagnetic waves with the same incident polarization and frequency remains a significant challenge due to the intrinsic properties of these waves.

To address this, new designs incorporating asymmetric transmission principles have been proposed. For example, Chen et al. presented a metasurface with vertical asymmetry [23], achieved through geometric rotation and torsion, which creates asymmetric transmission with the desired phase characteristics. While this approach disrupts vertical symmetry to enable different functions for opposite propagation directions, it still depends on distinct wave sources for different incidences and does not achieve full-space control with a single wave source. To overcome this limitation, introducing additional degrees of freedom is crucial for attaining effective and independent control.

As research on various active materials has advanced, materials such as graphene, vanadium dioxide, indium antimonide, and perovskites have been integrated into metasurface designs, enabling active control of electromagnetic waves. For instance, Lee et al. demonstrated an electrically tunable graphene-gold metasurface that enables active control of terahertz wave reflection. By modulating the Fermi level of graphene through electrostatic gating, they achieved dynamic switching between specular reflection and anomalous reflection, as well as beam steering capabilities [24]. Dicken et al. demonstrated a reconfigurable metasurface [25] based on vanadium dioxide, which undergoes a reversible phase transition from insulator to metal when heated above its critical temperature. By utilizing this property, they achieved dynamic control over the metasurface’s optical response, enabling switching between different functionalities. Sun et al. demonstrated that all-optical control of phase-change metamaterials [26] can achieve switching times on the order of femtoseconds. As a type of phase-change metamaterials, perovskites have attracted increasing attention due to their unique properties of functioning as quantum dots and generating two-dimensional electron gas, leading to their growing applications in metasurface fabrication [27,28,29]. Long et al. developed an all-dielectric perovskite metasurface design that overcomes traditional size mismatch and weak light-structure interaction limitations in chiral hybrid perovskites, achieving giant superstructural chirality through engineered planar nanostructures, demonstrating remarkable optical performance with an anisotropy factor of 0.49 and circular dichroism of 6350 mdeg through careful tuning of electric and magnetic multipole moments, while numerical simulations suggest potential for even higher optical activity with increased metasurface area [30]. With the advancement of perovskite research for metasurfaces, the process of patterning perovskite thin films has also become increasingly mature [31,32,33]. Incorporating perovskite into metasurface design significantly expands functional diversity.

In light of recent advancements, we propose a photoreconfigurable, multifunctional full-space terahertz metasurface. By harnessing the unique properties of perovskite materials, this metasurface enables efficient and independent control of both transmission and reflection modes through varying light intensities. Without laser illumination, it effectively transmits circularly polarized terahertz waves, and under specific light conditions, it reflects these waves to generate strong signals. Inspired by the ancient Chinese myth of Zhulong, a deity associated with controlling light and darkness, the metasurface uses light to manage terahertz waves in both modes. In transmission mode, the metasurface symbolizes brightness akin to daylight, while in reflection mode, it represents concealment similar to darkness. Its characteristics make it highly potential for dynamic imaging, offering real-time adjustment of imaging modes, image quality, and functionality. It also shows significant potential in communication systems, providing more efficient channel capacity and data transmission capabilities.

## 2. Theoretical Analysis and Design of Unit Structure

In this work, a design method for a photoreconfigurable full-space metasurface is proposed. In the absence of light, incident left circularly polarized (LCP) waves are transmitted and converted into right circularly polarized (RCP) waves. Under laser illumination with an intensity of 729.45 mW/cm^2^, incident LCP waves are reflected while maintaining the LCP state. We achieved independent control of the outgoing wavefront in both transmission mode (0 mW/cm^2^) and reflection mode (729.45 mW/cm^2^) with a single incident wave operating at an identical frequency.

**Figure 1:**
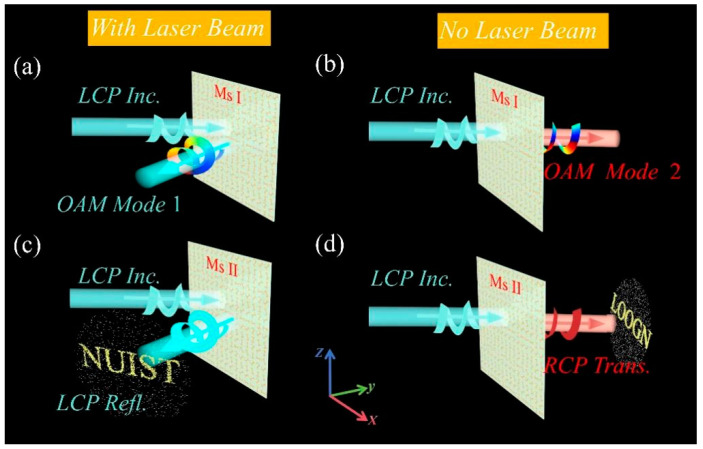
(**a**) OAM Mode 1 in transmission mode under high laser beam illumination for Metasurface I. (**b**) OAM Mode 2 in reflection mode without laser beam illumination for Metasurface I. (**c**) Holographic imaging with high laser beam illumination, generating a holographic image of the letters “NUIST” in reflection mode for Metasurface II. (**d**) Holographic imaging without laser beam illumination, generating a holographic image of the letters “LOONG” in transmission mode for Metasurface II.

Figure 1a,b illustrate the orbital angular momentum (OAM) modes of different orders realized by the proposed full-space metasurface under varying laser beam illumination. Specifically, without high laser beam illumination, OAM mode 2 is obtained in transmission mode, whereas under laser beam illumination, OAM mode 1 is achieved in reflection mode. By employing the metasurface convolution principle, the generated OAM beams are deflected in various directions, as evidenced by the reflection deflection in Figure 1a and the transmission deflection in Figure 1b. Additionally, Figure 1c,d showcase the dynamic imaging effects under varying laser beam illumination. Under high laser beam illumination, the metasurface generates a holographic image of the letters “NUIST” in reflection mode. In contrast, with no laser beam illumination, a holographic image of the letters “LOONG” is produced in transmission mode. The simulations demonstrated that the designed metasurface can efficiently and independently control the outgoing wavefront in both reflection and transmission modes, successfully achieving independent holographic imaging in various states.

To achieve the aforementioned functionalities, the designed metasurface unit cells must possess two critical characteristics: high-efficiency transmission and reflection at an identical frequency, and independent phase control capabilities in both transmission and reflection modes. Through rigorous design optimization and parameter tuning, we obtained the following metasurface unit cell structure.

**Figure 2:**
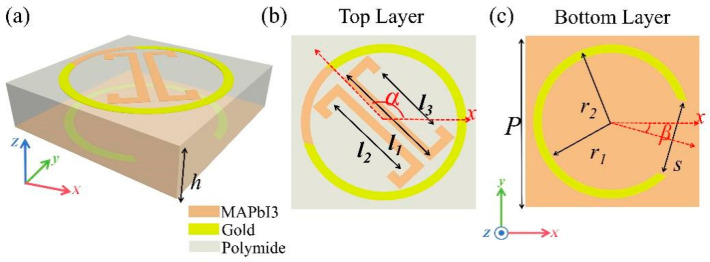
(**a**) Schematic of the proposed three-layer metasurface. (**b**) Top layer with CSRR and dual *C*-shaped resonators. (**c**) Bottom layer with circular split ring resonator.

The proposed metasurface, as shown in Figure 2a, consists of a three-layer structure. The top layer is composed of a circular split ring resonator (CSRR) structure with openings filled with perovskite material. At the center of the CSRR, dual *C*-shaped perovskite resonators are situated. The perovskite material [34,35], MAPbI3, serves as an active component. In the absence of laser beam illumination, it functions as an insulator with a dielectric constant of 33 and a conductivity of 0.08 S/m. When exposed to a 405 nm wavelength laser with an intensity of 729.45 mW/cm^2^, MAPbI3 undergoes a phase transition to a metallic state, exhibiting a dielectric constant of 60 and a conductivity of 1.4 × 10^7^ S/m. The middle layer is a dielectric layer made of polyimide, which has a dielectric constant of 3.5 and a loss tangent of 0.0027. This layer primarily serves as support and isolation, ensuring the effective coupling of electromagnetic waves between different layers. The bottom layer is a perovskite substrate with a CSRR cut out in the center, filled with gold, as shown in Figure 2b,c. The gold conductivity is set to 5.8 × 10^7^ S/m, with both the gold and perovskite thickness set to 1 μm. The other optimized parameters are as follows: polyimide dielectric layer height *h* = 27 μm, structure length and width *p* = 50 μm, circular split ring resonator outer radius *r*_1_ = 45 μm, inner radius *r*_2_ = 40 μm, and dual *C*-shaped resonator geometric parameters *l*_1_ = 66.89 μm, *l*_2_ = 56.60 μm, and *l*_3_ = 46.31 μm. At the same time, based on extensive research of existing fabrication techniques [31,32,33], we propose the following possible manufacturing process: The fabrication begins with preparing MAPbI3 perovskite solution from methylammonium iodide (MAI) and PbI2 in N-dimethylformamide (DMF)/dimethyl sulfoxide (DMSO) (7:3), followed by spin-coating under controlled conditions (relative humidity < 20%, temperature 22 ± 2 °C) in a nitrogen-filled glovebox. The dual *C*-shaped and CSRR structures are patterned through soft lithography using polydimethylsiloxane (PDMS) mold (50 kPa, 100 °C), while the gold structures are fabricated via e-beam evaporation and ion beam etching. Finally, after two-step annealing optimization (100 °C/10 min, 150 °C/15 min), a 27 μm polyimide protective layer is applied and cured (180 °C) in a nitrogen atmosphere.

For clarity in the description, the rotation angles are defined as follows: *α* represents the angle between the symmetry axis of the top layer’s dual *C*-shaped resonator and the *x*-axis, while *β* represents the angle between the symmetry axis of the CSRR and the *x*-axis. As shown in Figure 2b, the top layer’s dual *C*-shaped resonator is adjusted by rotation angle *α*, while the CSRR is adjusted by rotation angle *β*. This design enables the metasurface to achieve independent and precise control of phase responses in both reflection and transmission modes.

Considering specific laser beam illumination, particularly in reflection mode, the design of the proposed metasurface leverages the Pancharatnam–Berry (PB) phase principle [36,37,38,39]. This principle enables the continuous modulation of the phase response of orthogonal CP waves by altering the geometric orientation of the metasurface elements. The co-reflection coefficients of elements for LCP and RCP waves are described by the following equations [40]:

(1) $$r_{ll}=1/2\;[(r_{xx}-r_{yy})-j(r_{xy}+r_{yx})]exp(-j2\alpha )$$

(2) $$r_{rr}=1/2[(r_{xx}-r_{yy})+j(r_{xy}+r_{yx})]exp(+j2\alpha )$$

Here, *r_xx_* and *r_yy_* are the reflection coefficients for linearly *x* and *y* polarized waves, respectively, while *r_xy_* and *r_yx_* represent the cross-polarization reflection coefficients. The parameter *α* signifies the rotation angle of the element relative to the *x*-axis, as depicted in Figure 2b. When the element structures yield uniform amplitude responses for two orthogonal linearly polarized waves with a 180° phase difference (|*r_xx_*| = |*r_yy_*| = 1), the cross-polarization reflection coefficients (*r_xy_* and *r_yx_*) are effectively reduced to zero. Consequently, the CP incidence maintains a uniform reflection amplitude (|*r_l_*| = |*r_r_*| = 1). Furthermore, altering the rotation angle *α* of the elements induces an additional phase shift of ±2*α* in the reflected electromagnetic wave, with the “+” and “−” signs, respectively, corresponding to RCP and LCP incident waves.

In transmission mode, the PB phase metasurface operates similarly [36]. The transmission coefficients are described as:

(3) $$t_{lr}=1/2[(t_{xx}-t_{yy})-j(t_{xy}+t_{yx})]exp(-j2\beta )$$

(4) $$t_{rl}=1/2[{(t}_{xx}-t_{yy})+j(t_{xy}+t_{yx})]exp(+j2\beta )$$

Achieving |*t_xx_*| = |*t_yy_*| = 1 with a phase difference of *ϕ_xx_* − *ϕ_yy_* = ±π ensures complete transmission for CP waves (|*t_lr_*| = |*t_rl_*| = 1). The phase shift *ϕ* = ±2*β* provides full 360° phase control with high transmission efficiency. This ensures efficient CP wave manipulation in both reflection and transmission modes.

**Figure 3:**
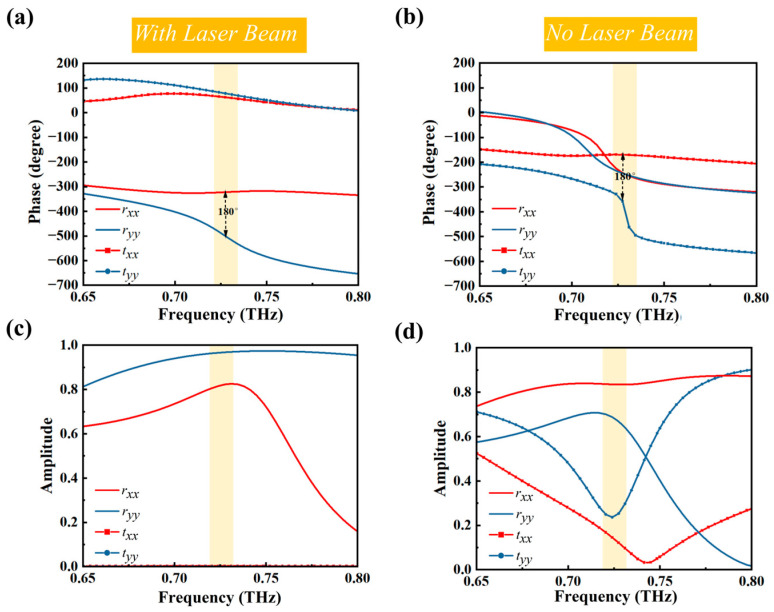
(**a**) Phase of co-polarized reflection coefficients (*r_xx_* and *r_yy_*) and co-polarized transmission coefficients (*t_xx_* and *t_yy_*) under laser beam illumination. (**b**) Phase of co-polarized reflection coefficients (*r_xx_* and *r_yy_*) and co-polarized transmission coefficients (*t_xx_* and *t_yy_*) without laser beam illumination. (**c**) Amplitude of co-polarized transmission coefficients (*t_xx_* and *t_yy_*) and co-polarized transmission coefficients (*t_xx_* and *t_yy_*) under laser beam illumination. (**d**) Amplitude of co-polarized transmission coefficients (*t_xx_* and *t_yy_*) and co-polarized transmission coefficients (*t_xx_* and *t_yy_*) without laser beam illumination.

As depicted in Figure 3a,c, the phase difference of the co-polarized reflection coefficients (*r_xx_* and *r_yy_*) under laser beam illumination exhibits a nearly 180° difference around 0.725 THz (as shown in yellow shaded region). The reflection amplitudes (|*r_xx_*| and |*r_yy_*|) approach unity, signifying effective operation within this frequency range. Additionally, the phase difference between *t_xx_* and *t_yy_* around 0.725 THz is nearly 0°, with their amplitudes also close to zero. According to Equations (1) and (2), the effective operating frequency range in reflection mode is around 0.725 THz under specific laser beam illumination, while the transmission mode is minimally operational at this frequency. Conversely, as shown in Figure 3b,d, in the absence of laser illumination, the metasurface works in transmission mode around 0.725 THz, with the reflection mode being minimally functional at this frequency.

**Figure 4:**
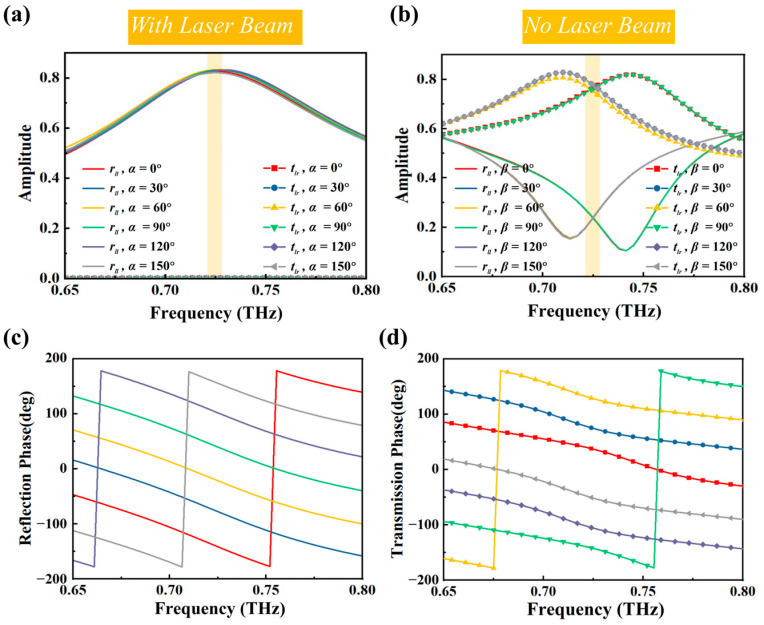
(**a**) Reflection amplitude and transmission amplitude at different rotation angles when unit is under laser beam illumination. (**b**) Transmission amplitude and reflection amplitude at different rotation angles when unit is without laser beam illumination. (**c**) Reflection phase at different rotation angles when unit is under laser beam illumination. (**d**) Transmission phase at different rotation angles when unit is without laser beam illumination.

The transmission and reflection amplitudes and phases of the unit for CP waves at different rotation angles are shown in Figure 4. As shown in Figure 4a, when the perovskite is in a metallic state, rotating the dual *C*-shaped structure maintains a nearly constant reflection coefficient, with a maximum value of 0.82 at 0.725 THz. As illustrated in Figure 4c, the reflection phase covers a 360° range by changing angle *α* across a wide bandwidth. Similarly, when the perovskite is in an insulating state, rotating the CSRRs maintains a high transmission coefficient near 0.725 THz, and the transmission phase also covers a full 360° range by changing angle *β*.

In summary, by controlling the external laser intensity and adjusting the rotation angle of the unit structure, independent control over the reflection and transmission properties of electromagnetic waves can be achieved.

**Figure 5:**
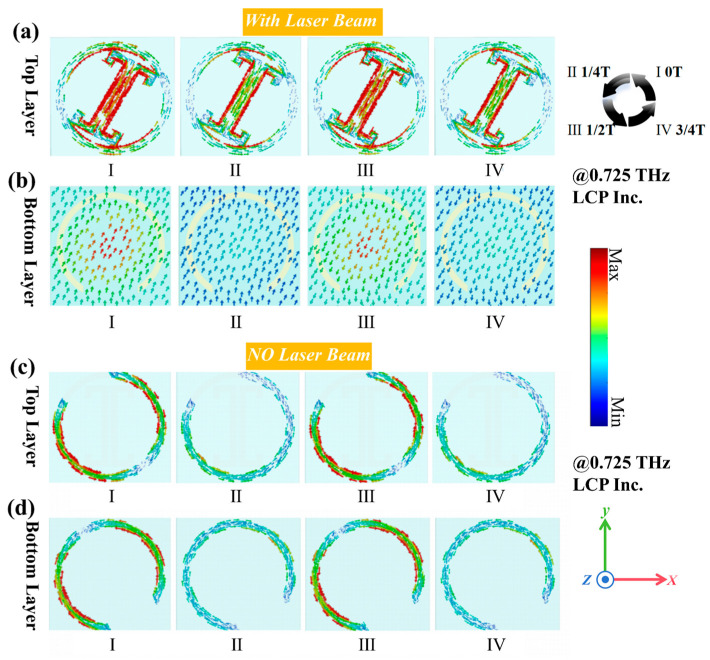
(**a**) Surface current distribution on the top layer under strong laser beam illumination, showing time-varying current distribution within the period. (**b**) Surface current distribution on the bottom layer under strong laser beam illumination, showing time-varying current distribution within the period. (**c**) Surface current distribution on the top layer without laser beam illumination, showing time-varying current distribution within the period. (**d**) Surface current distribution on the bottom layer without laser beam illumination, showing time-varying current distribution within the period.

The time-varying current distributions shown in Figure 5 demonstrate the working mechanism of our structure at *f* = 0.725 THz under the normal incidence of left-handed circularly polarized (LCP) waves. For one complete cycle (*T* = 1/*f* ≈ 1.38 ps), we selected four time points (*t* = 0, T/4, T/2, 3T/4) to reveal the periodic evolution of surface currents under different laser illumination conditions, which provides clear demonstration of the active structural components and their distinct electromagnetic responses in different modes.

To further elucidate the working mechanism of the proposed reconfigurable metasurface, the surface current distribution under laser beam illuminations and at various times was simulated, as shown in Figure 5a,b. Under strong laser illumination, the surface current on the top layer primarily concentrates on the dual *C*-shaped structure, while the surface current on the bottom layer of the metasurface is also strong, effectively acting as a complete metal plate [40,41]. Therefore, under laser beam illuminations, the dual *C*-shaped structure on the top layer and the metal plate on the bottom layer play a major role, being responsible for reflecting electromagnetic waves and manipulating the phase responses in reflection mode.

Similarly, the surface current distribution without laser beam illumination was simulated, as shown in Figure 5c,d. The surface current is distributed across the CSRRs on both the top and bottom layers at different times, with the current direction always opposite. This indicates that in transmission mode, the CSRRs are active, allowing the incident electromagnetic waves to be transmitted and efficiently modulating their phase and amplitude through coupling effects [36,41].

To evaluate the performance of our proposed metasurface, we compared its key characteristics with recent related works, as summarized in Table 1. Although [21,22] achieve higher transmission (87% and above 80%) and reflection amplitudes (92% and above 95%), our design incorporates additional critical features including frequency-preserving characteristics and switchable functionality. While [42] demonstrates similar comprehensive functionality with frequency-preserving property, independent phase control, and switchable operation, our work achieves notably higher transmission and reflection amplitudes (82% and 80% compared to above 60% and approximately 50%). Compared to [43], which shows excellent transmission and reflection performance (above 86% and 85%) with frequency-preserving and switchable properties, our work maintains comparable amplitude performance while also offering independent phase control capability. Our design achieves superior performance compared to [19] by incorporating all three key functionalities (frequency-preserving, independent phase control, and switchable operation) while maintaining high transmission and reflection amplitudes (82% and 80%).

Overall, our proposed metasurface uniquely combines all desired characteristics-frequency-preserving property, independent phase control, and switchable operation while maintaining high transmission (82%) and reflection (80%) amplitudes, demonstrating a well-balanced approach to full-space metasurface design.

## 3. Wavefront Manipulation Applications

### 3.1. Full-Space OAM Generator

A vortex beam is a type of beam that has an annular intensity profile and a helical phase front, carrying OAM. Generating vortex beams is a crucial application in electromagnetic wave manipulation, significantly improving spectrum utilization and increasing information capacity in wireless communications. Due to the infinite modes of orthogonal information channels provided by OAM, multifunctional OAM vortex beams based on metasurfaces have broad application prospects in modern communication systems. Our proposed reconfigurable metasurface can generate vortex beams of different orders with distinct deflected directions in both transmission and reflection modes. The phase equation of the vortex beam’s wavefront is *Φ* = *e^jlφ^*, where *l* is the topological charge or mode number of the vortex beam, and *Φ* is the azimuthal angle, the required phase of the metasurface at (*x*, *y*) is given by [44].

(5) Φ(x,y,l)=l*arctan(y/x)

**Figure 6:**
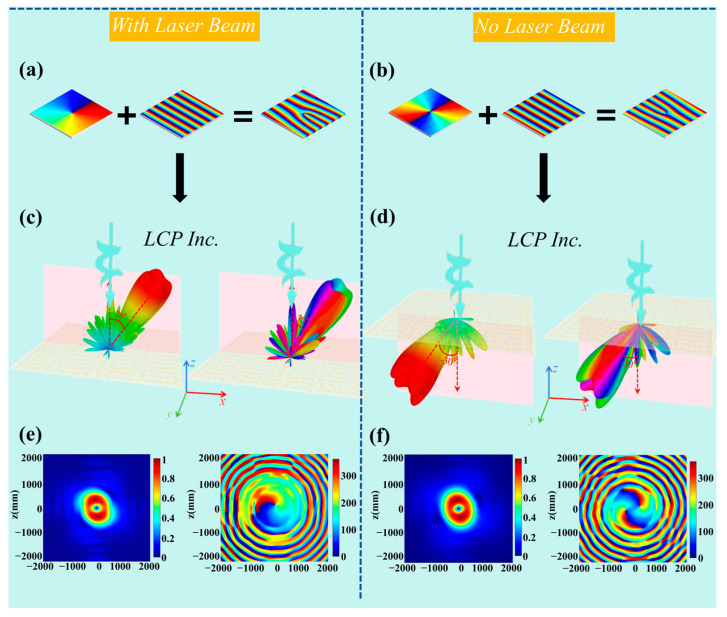
(**a**) Phase gradients along the +*x* direction. (**b**) Phase gradients along the −*x* direction. (**c**) Far-field distribution of a +1 order vortex beam deflected by 30° in the +*x* direction under strong laser beam illumination. (**d**) Far-field distribution of a −2 order vortex beam deflected by 30° in the −*x* direction without laser beam illumination. (**e**) Planar electric field intensity and phase distribution of the +1 order vortex beam under strong laser beam illumination, perpendicular to the 30° direction. (**f**) Planar electric field intensity and phase distribution of the −2 order vortex beam without laser beam illumination, perpendicular to the −30° direction.

The reconfigurable metasurface array, composed of 48 × 48 unit structures, was excited by LCP plane waves. Figure 6a,b display the phase distributions for the vortex beam generator when the topological charges are *l* = +1 and *l* = +2, respectively. These phase distributions represent the initial states without deflection. According to the metasurface convolution principle [45], vortex beams directed in any spatial orientation can be generated. Figure 6a,b also show the phase gradients required to deflect the beams by 30° along both the +*x* and −*x* directions. After convolving the phases carrying +1 and +2 order OAM with the phase gradients required for deflection of 30°, the final phase distributions for the beams deflected along the +*x* direction by +1 order OAM and along the −*x* direction by −2 order OAM are obtained.

In Figure 6c, the simulation results reveal the far-field distribution of a +1 order vortex beam deflected by 30° in the +*x* direction under strong laser illumination. This beam exhibits a clear directional deflection and distinct helical wavefront characteristics in the reflection mode. Conversely, Figure 6d illustrates the far-field distribution of a −2 order vortex beam deflected by 30° in the −*x* direction without laser illumination.

Furthermore, Figure 6e,f depict the planar electric field intensity and phase distribution of the +1 order and −2 order vortex beams, respectively, perpendicular to the 30° and −30° directions. Under conditions of strong laser illumination and no illumination, the amplitude maps indicate that the ring structure and intensity distribution are consistent with the characteristics of vortex beams. The phase maps clearly show the helical phase distributions of the 1st and 2nd order vortex beams, further verifying their generation.

Utilizing photoreconfigurable metasurfaces to generate vortex beams can significantly enhance spectrum utilization and information capacity in wireless communications. By generating vortex beams of different orders and directions in both transmission and reflection modes, multifunctional OAM vortex beams can be applied. This technology can provide more efficient channel capacity and data transmission capabilities in communication systems.

### 3.2. Full-Space Holographic Imaging

Traditional holography relies on the interference between reference light and the scattered waves from an object. Digital holography, on the other hand, involves calculating holographic interference patterns (including phase and amplitude information) and encoding them onto surface structures such as metasurfaces or spatial light modulators (SLMs) to generate holographic images. Compared to traditional holography, computational holographic imaging uses numerical simulations to model the holographic surface, offering greater design flexibility and allowing for the optimization of imaging algorithms to enhance image quality, eliminate redundant diffraction components, and improve imaging efficiency. Because metasurfaces can precisely manipulate electromagnetic wavefronts, many studies focus on generating holograms using metasurfaces. This study employs metasurfaces with full-space phase manipulation capabilities to achieve super-holography in both transmission and reflection modes.

The design of the holographic system consists of two main steps: using holographic algorithms to numerically calculate the required phase distribution and using the metasurface to construct the phase array. In computational holography design, the diffraction between the metasurface and the imaging plane’s electromagnetic field is a reversible problem. According to the Rayleigh–Sommerfeld diffraction formula, each unit on the metasurface holographic plate radiates an electromagnetic field, and the unit’s radiated field can be superimposed on the imaging plane. First, we assume that the radiated electric field of the metasurface is [46]:

(6) $$U(x_{0},y_{0})=\frac{1}{j\lambda }\iint_{s}U(x,y)\cos\langle n,r\rangle \frac{\exp(jkr)}{r}dS$$

where *U* (*x*_0_, *y*_0_) and *U* (*x*, *y*) are the electric field distributions on the metasurface plane and the imaging plane, respectively, and $r=\sqrt{\left[{\left(x-x_{0}\right)}^{2}+{\left(y-y_{0}\right)}^{2}+{z_{d}}^{2}\right]}$ is the distance between any two points on the imaging plane and the metasurface. *λ* is the wavelength, and *z_d_* is the distance between the imaging plane and the metasurface. Since we use a pure phase holography method in this paper, we set the amplitude to 1, and the electric field on the imaging plane can be calculated as [47]:

(7) $$U^{\prime }(x,y)=\frac{1}{j\lambda }\iint_{s_{0}}U(x_{0},y_{0})\cos\langle n,r\rangle \frac{\exp(-jkr)}{r}dS_{0}$$

$U^{\prime }(x,y)$ is replaced with the amplitude of the image as the input for the calculation. To achieve better imaging quality, we use the Gerchberg–Saxton (GS) iterative algorithm to optimize the calculated holographic phase [47]. After multiple iterations, the electric field on the hologram is derived, and the amplitude and phase information is extracted to construct the hologram.

**Figure 7:**
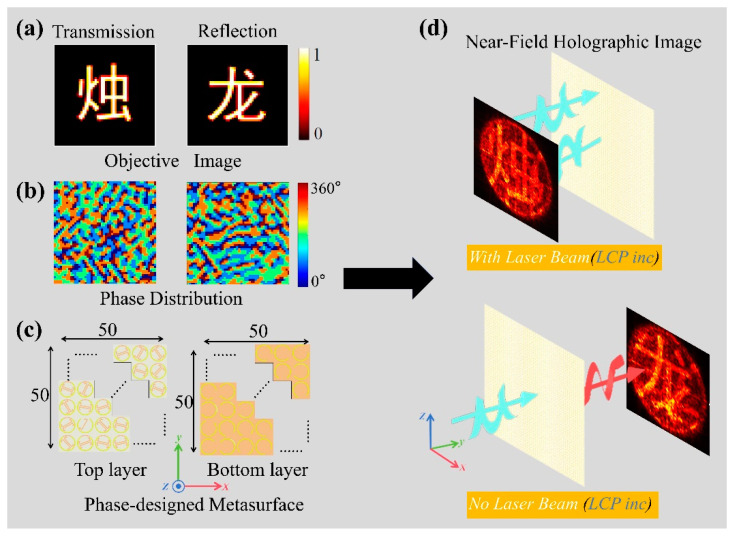
(**a**) Target images: “Zhu” in reflection mode, “Long” in transmission mode. (**b**) Phase distribution for holographic images, with phase changes from 0° to 360°. (**c**) Metasurface design layout with 50 × 50 unit structures in top and bottom layers. (**d**) Simulation results under different laser beam illumination: “Zhu” in reflection mode under laser beam illumination; “Long” in transmission mode without laser beam illumination.

Using the proposed metasurface unit structure, we constructed holograms in both reflection and transmission modes under different light intensities. The designed metasurface holographic plate consists of 50 × 50 unit structures, with a size of 5 mm × 5 mm. The design process is shown in Figure 7.

First, the Chinese characters “Zhu” and “Long” were selected as target images for the holographic design, as shown in Figure 7a. The image of the character “Zhu” is generated in reflection mode, while the image of the character “Long” is generated in transmission mode. Using the GS algorithm, the phase distributions required to achieve these two holographic patterns were calculated, as shown in Figure 7b. Based on the phase distribution, the proposed metasurface was constructed, with the top and bottom layer structures illustrated in Figure 7c. During the simulation process, LCP plane waves were used as the incident waves, and an observation plane was placed 2 mm away from the metasurface. Figure 7d shows that the holographic image of the character “Zhu” is generated in reflection mode under strong laser beam illumination. Meanwhile, the holographic image of the character “Long” is generated in transmission mode without laser beam illumination.

**Figure 8:**
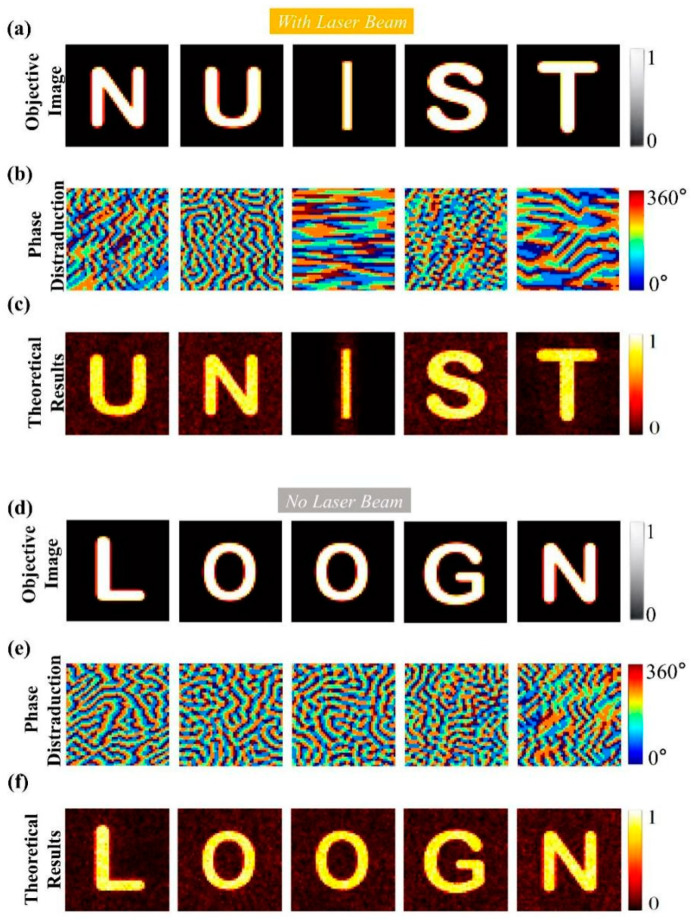
(**a**) Target images in reflection mode. (**b**) Phase distribution for reflection mode calculated using the GS algorithm. (**c**) Reproduced image of “NUIST” in reflection mode. (**d**) Target images in transmission mode. (**e**) Phase distribution for transmission mode calculated using the GS algorithm. (**f**) Reproduced image of “LOOGN” in transmission mode.

To further illustrate, another example of two English words was used. In the reflection mode, the letters “NUIST” were selected as imaging targets, while in the transmission mode, the letters “LOOGN” were chosen. Based on the theoretical description above, the GS algorithm was utilized to optimize the phase distribution of the metasurface, resulting in the final phase distribution. Figure 8 provides a comprehensive illustration of this process. Figure 8a,d present the target images for the reflection and transmission modes, respectively. The phase distributions of the metasurface, calculated by the GS algorithm, are shown in Figure 8b,e for the reflection and transmission modes, respectively. Figure 8c,f display the theoretical calculated images corresponding to phase distributions in Figure 8b,e: “NUIST” in reflection mode and “LOOGN” in transmission mode. The theoretical results show that the phase distributions optimized using the GS algorithm effectively generate the desired target images, which meets expectations.

The final constructed metasurface is 25 mm by 5 mm, and its imaging on the observation plane was simulated. An LCP wave at a frequency of 0.725 THz was used as the incident source, and the outgoing electric field was extracted from the reflection plane and the transmission plane, each positioned 1 mm away from the metasurface.

**Figure 9:**
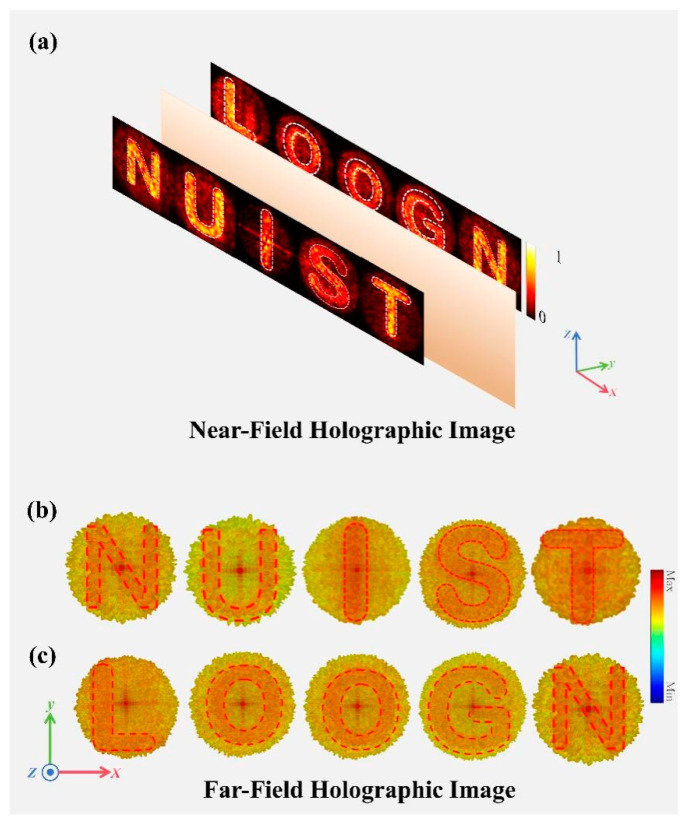
(**a**) Near-field imaging results. (**b**) Far-field electric field distribution in reflection mode. (**c**) Far-field electric field distribution in transmission mode.

Figure 9a shows the simulated near-field imaging results. In the reflection mode, the electric field distribution clearly reveals the letters “NUIST”. Similarly, in the transmission mode, the electric field distribution presents the letters “LOOGN”. These near-field results demonstrate that the proposed metasurface effectively generates the desired holographic patterns in both modes. Further analysis in the far field confirms these results. Figure 9b depicts the far-field electric field distribution in reflection mode, where the letters are distinctly visible. This observation verifies the metasurface’s capability to accurately image in reflection mode. Similarly, the far-field electric field distribution in transmission mode is shown in Figure 9c. Here, the letters are clearly identifiable, proving the metasurface’s imaging effectiveness in transmission mode as well.

These simulation results indicate that the proposed reconfigurable full-space metasurface can effectively achieve independent holographic imaging in both reflection and transmission modes under different laser beam illumination. By adjusting the laser beam intensity, the metasurface can freely switch between modes to generate various holographic patterns. This reconfigurability allows for real-time adjustment of imaging modes, image quality, and functionality in dynamic imaging. Furthermore, its operation in the terahertz frequency range makes it highly suitable for high-resolution imaging applications.

## 4. Conclusions

This study presents a photoreconfigurable multifunctional full-space terahertz metasurface based on perovskite materials. Inspired by the ancient Chinese myth of Zhulong, which controls light and darkness, we designed a metasurface structure capable of switching between transmission and reflection modes through variations in laser beam intensity. This innovative design demonstrates novel full-space wavefront manipulation capabilities. As validation, we achieved +1 order and +2 order vortex beams in transmission and reflection modes, respectively, and used the metasurface convolution principle to achieve vortex beam deflection in different directions. Utilizing metasurface holographic imaging technology, we achieved dynamic imaging in both spaces, generating the letter patterns “NUIST” in the reflection space and “LOOGN” in the transmission space. Simulation results indicate that the proposed reconfigurable metasurface device can achieve excellent wavefront manipulation effects in both transmission and reflection modes. Our work offers a reference for designing efficient dynamically reconfigurable full-space metasurfaces. The proposed metasurface has potential applications in dynamic imaging and communication systems, enabling reconfigurable adjustments.

## Figures and Tables

**Table 1:** Performance comparison of related works.

Ref.	Freq- Preserving	Independent Phase Control	Switchable	Transmission Amplitude	Reflection AMPLITUDE
**[19]**	**×**	**×**	**×**	above 60%	above 70%
**[21]**	**×**	√	**×**	above 80%	above 95%
**[22]**	**×**	√	**×**	87%	92%
**[42]**	√	√	√	above 60%	approximately 50%
**[43]**	√	**×**	√	above 86%	above 85%
**Our work**	√	√	√	82%	80%

## Data Availability

Data underlying the results presented in this paper are not publicly available at this time but may be obtained from the authors upon reasonable request.
